# Targeted Therapies for Pancreatic Cancer

**DOI:** 10.3390/cancers10020036

**Published:** 2018-01-29

**Authors:** Idoroenyi Amanam, Vincent Chung

**Affiliations:** Department of Medical Oncology & Therapeutics Research, City of Hope Comprehensive Cancer Center, Duarte, CA 91010, USA; iamanam@coh.org

**Keywords:** targeted therapy, personalized medicine, pancreas

## Abstract

Pancreatic cancer is the third leading cause of cancer related death and by 2030, it will be second only to lung cancer. We have seen tremendous advances in therapies for lung cancer as well as other solid tumors using a molecular targeted approach but our progress in treating pancreatic cancer has been incremental with median overall survival remaining less than one year. There is an urgent need for improved therapies with better efficacy and less toxicity. Small molecule inhibitors, monoclonal antibodies and immune modulatory therapies have been used. Here we review the progress that we have made with these targeted therapies.

## 1. Introduction

Despite recent advances in chemotherapy, pancreatic cancer remains a deadly disease and is the third leading cause of cancer related death in the United States [1]. Only about 25% of patients are surgical candidates at the time of diagnosis and even after surgical resection, only about 20% of those patients live longer than 5 years. With the majority of patients presenting with unresectable disease, chemotherapy is the mainstay of treatment. Over the last 15 years, we have made incremental progress in improving overall survival. Gemcitabine is a standard treatment with a 5–10% response rate and average median overall survival of 6 months. This study showed a 24% clinical benefit and is commonly used in patients with poor performance status [2]. In 2005, the National Cancer Institute (NCI) Canada trial showed a 2-week improvement in median overall survival with gemcitabine and erlotinib [3]. This was the first targeted therapy approved for the treatment of pancreatic cancer but with only marginal benefit. In 2010, FOLFIRINOX treatment nearly doubled median overall survival compared to gemcitabine chemotherapy; however, at the cost of increased toxicity [4]. The most widely used chemotherapy regimen is gemcitabine and nab-paclitaxel due to its favorable toxicity profile even though median overall survival was less than FOLFIRINOX chemotherapy [5]. With these small incremental gains, there is an urgent unmet need for better and less toxic treatments.

In the last 15 years, there has been a paradigm shift in the treatment of solid tumors from traditional cytotoxic chemotherapies to more targeted therapies. Utilizing genomic analysis for molecular profiling of tumors, we have been able to discover driver mutations leading to cell proliferation and tumor metastasis. We have seen in Epidermal Growth Factor Receptor (EGFR) mutated lung cancer that targeting with a small molecule inhibitor of EGFR can lead to impressive response rates and tumor control better than standard cytotoxic chemotherapy. Unfortunately, we have not had the same successes with pancreatic cancer. In 2008 Jones et al. published results of a comprehensive genetic analysis of 24 pancreatic cancers. They determined the sequence of 23,219 transcripts representing 20,661 protein coding genes and found that pancreatic cancer contained on average 63 genetic alterations with a core set of 12 cellular signaling pathways that were altered [6]. Since then there have been many clinical trials targeting these altered pathways and here we review some of these approaches.

## 2. Pathogenesis of Pancreatic Cancer

**Figure 1:**
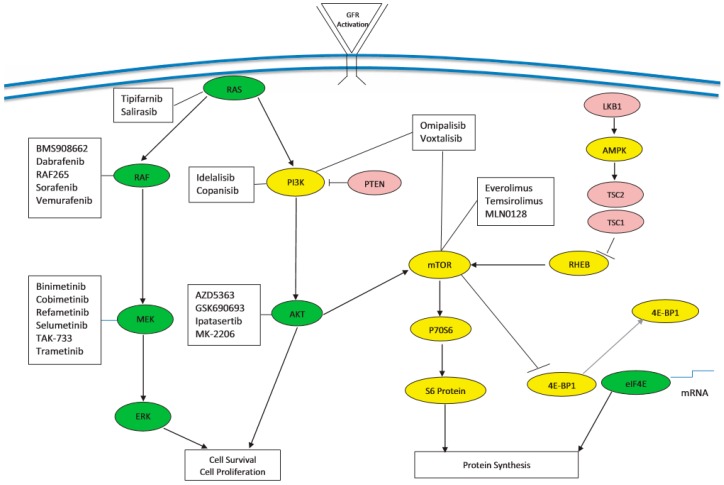
Illustration of Rat sarcoma (RAS) and phosphatidylinositiol 3-kinase (PI3K) Signaling. Redundant pathways allow for continued signaling leading to cellular proliferation and survival. A combinatorial approach with agents may overcome resistance.

The development of pancreatic cancer is usually in a stepwise fashion arising mainly from pancreatic intraepithelial neoplasia. Kirsten rat sarcoma (KRAS) mutation has been found to be an initiating genetic event for the majority of pancreatic ductal adenocarcinomas, with 95% of pancreatic intraepithelial neoplasms harboring KRAS mutations on chromosome 12 [7]. This proto-oncogene was first identified in Kristen rat sarcoma virus and performs an essential function of normal tissue signaling. Mutation in this gene acts like it on switch recruiting additional signaling proteins necessary for cellular proliferation. Tumor suppressor genes play an important role in preventing the growth of tumors and there are 3 commonly mutated ones found in pancreatic cancer. Cyclin-dependent kinase Inhibitor 2A (CDKN2A) is mutated or its promoter methylated in 95% of pancreatic tumors [8]. This encodes for p16/Ink4a and p14/Arf, which are inhibitors of Cyclin-dependent kinase 4/6 (CDK4/6) and Mouse double minute 2 homolog (MDM2) mediated p53 tumor suppressor degradation [9,10]. This CDK4/6 hyperactivation then leads to inactivation of the RB tumor suppressor leading to additional proliferation [9,11]. In later stages, there is loss of function in the tumor suppressor p53 which is mutated in 75% of PDAC [12]. P53 is the guardian of the genome and normal function leads to apoptosis of cancer cells. Mothers against decapentaplegic homolog 4 (SMAD4) mutation is associated with loss of SMAD4 protein expression. SMAD4 normally mediates signals from the family at TGF beta ligands which plays a dual role of either cellular proliferation or cellular apoptosis. It is mutated in approximately 55% of pancreatic adenocarcinomas [13]. Due to aberrant autocrine and paracrine signaling, multiple pathways leading to cellular proliferation, migration and invasion are activated by signaling molecules such as a hepatocyte growth factor, fibroblast growth factor and insulin like growth factor 1. Many trials have been completed trying to target these various pathways but have been unsuccessful (Figure 1). 

## 3. Transmembrane Receptor Proteins

Epidermal growth factor receptor is a member of the ErbB family of tyrosine kinases including ErbB1/EGFR, ErbB2/HER2, ErB3 and ErbB4. EGFR is a transmembrane glycoprotein containing an extracellular N-terminal ligand binding domain, transmembrane region and a C-terminal intracellular domain with phosphorylation sites. Binding of the ligand to the receptor leads to dimerization and auto-phosphorylation, activating the RAS/mitogen activated protein kinase as well as the phosphatidyl inositol 3 kinase/AKT pathways [14,15]. Since a majority of pancreatic cancers overexpress EGFR, there was significant interest in targeting this pathway. The National Cancer Institute of Canada Clinical Trials Group (NCIC CTG) coordinated a large randomized phase 3 clinical trial comparing gemcitabine versus gemcitabine with erlotinib, a small molecule tyrosine kinase inhibitor which competes for the ATP binding site on the intracellular domain. This was done in an unselected patient population and showed an improvement in median overall survival with the combination of 6.24 months versus 5.91 months. The hazard ratio was 0.82 and this regimen was approved by the Food and Drug Administration (FDA) as a standard treatment for patients with advanced pancreatic cancer [3]. The bar for approval was very low since many negative clinical trials preceded this one. Efforts were made to improve on these results utilizing other EGFR targeting agents.

Cetuximab is a chimeric monoclonal antibody which binds to the epidermal growth factor receptor on the extracellular surface preventing ligand binding. This has a direct anti-proliferative effect on the tumor cell decreasing signal transduction leading to G1 cell cycle arrest and apoptosis. The Fc region of the antibody also permits the host immune system to recognize the antibody coated tumor cell and destroy it. Based upon smaller phase 2 studies, the combination appeared promising and the Southwest Oncology Group conducted a large randomized phase 3 trial of cetuximab in combination with gemcitabine versus gemcitabine alone. 745 eligible patients were accrued and tissue was collected to study tumoral EGFR expression. There was no difference in survival seen between the 2 arms with a median overall survival 6.3 months for gemcitabine plus cetuximab versus 5.9 months for gemcitabine alone. The objective response rate and progression free survival were similar in both arms. For patients with available tumor tissue, tumoral EGFR expression was seen in 90% of the samples; however, this was not associated with a treatment benefit in this subset population [16].

**Figure 2:**
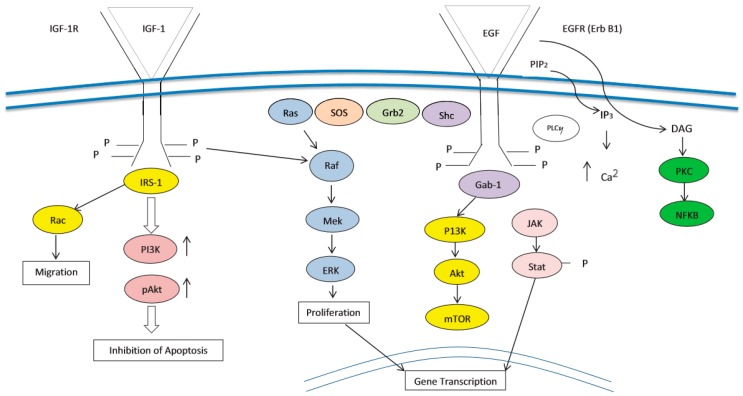
Convergence of IGFR and EGFR Signaling. Rationale for dual blockade of the receptors.

Insulin like growth factor 1 receptor is a transmembrane protein activated by the hormone insulin like growth factor 1. High levels of insulin-like growth factor-1 (IGF-1) have been associated with poor prognosis [17]. This has been implicated in several cancers and confers resistance to EGFR inhibitors by forming a heterodimer with the receptor allowing continued signaling. Preclinical studies suggested that simultaneously targeting EGFR and IGFR pathways would result in more effective growth inhibition and apoptosis since there was crosstalk leading to downstream signaling of pathways shared by both receptors (Figure 2). SWOG conducted a clinical trial S0727 which was a phase 1B and randomized phase 2 study of gemcitabine, erlotinib and cixutumumab versus gemcitabine plus erlotinib. 116 patients were randomized in the phase 2 portion of the study and the primary endpoint was Progression-free survival (PFS). The triple combination therapy was associated with higher incidences of elevated transaminases, fatigue, gastrointestinal effects, neutropenia and thrombocytopenia. The skin toxicity appeared to be similar amongst both arms. Unfortunately, there was no difference in median progression free survival and overall survival. Additional novel agents are being explored. MM-141, a bispecific antibody prevents PI3K/AKT/mTOR activity by blocking IGF-1R and ErbB3. Data from the phase 1 study of MM-141 was biomarker driven and showed that those with high free serum IGF-1R levels were able to stay on therapy twice as long (3.2 cycles vs. 1.8 cycles) [18]. Currently there is a phase 2 study testing MM-141 in combination with gemcitabine and nab-paclitaxel (NCT02399137). 

The results of these trials were likely impacted by the fact that most pancreatic cancer patients are KRAS mutated. In colorectal cancer, only KRAS wild type patients are able to receive therapy with EGFR inhibitors. There has been renewed interest in exploring the small subset population of KRAS wild type pancreatic cancer patients. A randomized phase IIB study of gemcitabine with or without nimotuzumab was recently reported in the Annals of Oncology. Nimotuzumab is a humanized IgG1 monoclonal antibody to the extracellular domain of EGFR with a favorable toxicity profile due to higher accumulation in tissues with higher EGFR expression. This study randomized 196 patients with KRAS wild type locally advanced or metastatic pancreatic cancer to 1 of 2 arms. 186 patients were evaluable for efficacy and safety. The medium overall survival and progression free survival for the experimental arm was 8.6 and 5.1 month respectively. In the control arm it was 6 months and 3.4 months respectively [19]. Even though these early studies appear promising, we are hitting the ceiling with survival being less than 1 year.

Vascular endothelial growth factor (VEGF) receptor is a transmembrane protein with an intracellular tyrosine-kinase domain. Binding to the VEGF receptor by ligand leads to dimerization and activation of signaling proteins which stimulate the formation of blood vessels. This allows tumors to get adequate blood supply to continue proliferation. Overexpression of VEGF is commonly seen in malignancies and is associated with a poor prognosis. Pancreatic cancer is not grossly vascular but studies have shown a correlation with blood vessel density, tumor VEGF-A levels and disease progression [20]. Preclinical studies showed that blocking VEGF resulted in regression of tumors [21]. Tumor vasculature is disorganized and hyperpermeable resulting in increased interstitial fluid pressure which impaired the delivery of chemotherapy. Treatment with a VEGF inhibitor led to normalization of the vasculature with improved delivery of chemotherapy. A phase 2 trial with gemcitabine and bevacizumab, a monoclonal antibody targeting VEGF-A, appeared promising with an objective response rate of 21% and a median overall survival of 8.8 months. This led to the phase 3 CALGB 80,303 trial which randomized 535 patients to either gemcitabine + placebo (GP) or gemcitabine + bevacizumab (GB). Unfortunately, there was no difference in overall survival (5.9 months GP versus 5.8 months GB *p* = 0.95) [22]. Small molecule inhibitors of VEGF such as axitinib have also been unsuccessful. A small 56 patient randomized phase 2 study exploring maintenance sunitinib after first line chemotherapy had encouraging results. This trial showed an improvement in 2-year overall survival, 7.1% for the observation arm and 22.9% for the sunitinib arm [23]; however, a large phase 3 trial would need to be done to confirm the results. Currently, there are on-going studies to see if the combination of VEGF inhibitors with more aggressive cytotoxic regimens would be beneficial.

## 4. RAS, the Elusive Target

With greater than 90% of pancreatic ductal adenocarcinoma’s harboring a KRAS mutation, targeting the RAS signaling pathway is an obvious but difficult approach. For over 3 decades, there have been multiple approaches to try to target RAS by effecting its activation and downstream signaling. After translation, KRAS is farnesylated allowing the protein to associate with the plasma membrane and associated activating proteins [24,25]. KRAS then interacts with SOS assisting KRAS binding to GTP resulting in activation. It was thought that farnesyltransferase inhibitors (FTIs) were the silver bullet to target KRAS by preventing its proper functioning. A phase III randomized, double blind, placebo controlled study comparing gemcitabine plus tipifarnib versus gemcitabine plus placebo was conducted and 688 patients were enrolled. Patients with advanced unresectable pancreatic cancer were eligible and the primary endpoint study was overall survival. The experimental arm and a higher incidence of grade 3 or higher neutropenia and thrombocytopenia although the toxicities were manageable. Unfortunately, there was no difference in overall survival, 193 versus 182 days [26]. Farnesyltransferase inhibitors did not appear to have an effect on pancreatic cancer cellular proliferation but there may be other beneficial effects of reducing pro-inflammatory cytokine secretion which plays an important role in the tumor microenvironment.

Another method to block Ras signaling is to interfere with the spacio-temporal localization of the proteins in the membrane. KRAS is aided in translocation to the membrane by the prenyl-binding protein phosphodiesterase 6 delta (PDEδ). Zimmerman, et al. saw this as an opportunity to suppress oncogenic RAS signaling by altering its localization to endomembranes. They performed high throughput screening to optimally select a small molecule inhibitor to bind to the pocket with nano-molar affinity. Proliferation of pancreatic ductal adenocarcinoma cells were inhibited in vitro and in vivo with deltarasin, which inhibits the PDEδ-KRAS interaction [27]. Salirasib, a Ras farnesylcysteine mimetic, dislodges Ras from the cell membrane and has been studied in combination with gemcitabine [28]. It has shown potential as a KRAS inhibitor in preclinical and clinical data [29]. Early-phase studies determined a safe dose of salirasib in combination with gemcitabine. Larger studies with robust biomarkers will be needed to evaluate the effectiveness of this therapy (Table 1).

Targeting downstream pathways is another approach. RAS-GTP preferentially binds to RAF, resulting in translocation of RAF to the plasma membrane. Active RAF phosphorylates and activates the MEK1 and MEK2 kinases which in turn activate ERK1 and ERK2. It has been shown in KRAS mutant tumors, that RAF inhibition may lead to paradoxical activation of ERK [30] by RAF dimerization leading to activation of CRAF. Mutant RAS also activates PI3K binding to PIP2 and phosphorylating it to PIP3 leading to activation and phosphorylation of AKT. MTOR is then activated leading to growth factor signaling, cell growth and proliferation. The PI3K/PTEN/Akt/mTORC1 is a key pathway activated in pancreatic cancer, likely due to its association with KRAS [31]. Monotherapy targeting PI3K, AKT and mTOR have not been successful in RAS mutant pancreatic cancer. PI3K pathway inhibition when combined with RAF-MEK-ERK inhibition is currently under investigation. A randomized phase II study evaluating selumetinib, a MEK inhibitor and MK-2206, an AKT inhibitor failed to show any benefit compared to mFOLFOX in patients who failed gemcitabine based therapy [32]. In order to potentially be effective, this required continuous daily dosing of the targeted therapy. Due to the overlapping toxicities of the small molecule inhibitors, patients were not able to stay on standard doses. With dose delays and reductions, the therapy was not able to sustain target inhibition and thus was not effective (Table 2).

## 5. Targeting the Tumor Microenvironment

The microenvironment of pancreatic cancer is characterized by a desmoplastic reaction caused by a heterogeneous group of cells. Pancreatic stellate cells are important in the modeling of normal tissue by producing metalloproteinases which assists in modifying the extracellular matrix but its activation by cytokines or other soluble factors also leads to fibrosis and increased intratumoral pressures preventing delivery of chemotherapy as well as creating an inhospitable environment for immune cells [36]. These stromal elements contribute to tumor growth and aggressiveness [37].

Hyaluronan is a naturally occurring nonsulfated glycosaminoglycan that primarily forms the extracellular matrix. Normal connective, neural and epithelial tissues contain hyaluronan but in malignancies, high levels have been associated with poor prognosis with accelerated tumor growth and decreased survival. Due to the high interstitial pressures observed in tumors which impacted perfusion, many groups were interested in breaking down this barrier with pegylated hyaluronidase (PEGPH20). In the phase II study combining gemcitabine, nab-paclitaxel and PEGPH20, there was not difference seen in survival for an unselected population [38]. Also, due to the ubiquitous nature of hyaluronan, there were unexpected toxicities. The FDA placed a clinical hold on the study when unexpected arterial and venous thrombosis was observed. This required amending the study to require anticoagulation with lovenox to prevent life threatening blood clots. The SWOG study with FOLFIRINOX and PEGPH20 initially only used aspirin prophylaxis. This proved to be inadequate and the study had to be amended to require lovenox. The data and safety monitoring committee closed the study before completion due to lack of activity. For the gemcitabine, nab-paclitaxel and PEGPH20 study, subset analysis was performed on the HA high patients. In the arm receiving PEGPH20, the response rate was 45% compared to 31%. With these encouraging results, the phase III HALO 301 study was designed as the largest biomarker driven trial evaluating HA high patients who receive gemcitabine and nab-paclitaxel with or without PEGPH20 (NCT02715804).

## 6. Targeting the Cancer Stem Cell

Survival of pancreatic cancer patients has remained poor despite improvements in chemotherapy. Previous studies have elucidated a population of resistant cells that are unable to be eradicated by most drugs leading to tumor relapse and metastasis. Less than 1% of the cells represent the cancer stem cell characterized by the markers CD24, CD44, CD133, CXCR4, ESA and nestin [39]. The hedgehog pathway plays an important role in the maintenance of cancer stem cells as well as activating pancreatic stellate cells and regulating the stroma [40]. This pathway is normally active during embryogenesis and turns off at birth but tumor cells increase the production of Hh ligand [41,42] which binds to the PTCH1 receptor leading to internalization and degradation of SMO. Transcription factors GLI1 and GLI2 are translocated to the nucleus inducing transcription of certain genes including ECM proteins [43]. Cyclopamine was the first SMO antagonist noted to have activity in preclinical studies [44,45]. In pancreatic cancer cell lines, treatment with cyclopamine resulted in down regulation of snail and up-regulation of E-cadherin consistent with inhibition of epithelial-to-mesenchymal transition. Combination with gemcitabine was effective in eradicating metastasis and shrinking the primary in orthotopic models. The preclinical data looked very compelling leading to the development of many trials. Vismodegib, a second generation cyclopamine approved for advanced basal cell carcinoma, was tested in a phase II clinical trial in combination with gemcitabine. In this double-blind trial, 106 patients were randomized and the primary endpoint was PFS. The combination arm had a PFS of 4 months while the gemcitabine arm was 2.5 months. The overall survival was similar at 6.9 months and 6.1 months respectively which was not statistically significant [46]. Saridegib (IPI-926) had a shocking turn of events when their randomized phase 2 clinical trial showed a worse survival with the combination compared to gemcitabine alone [47]. There was some speculation that the breakdown of the stroma allowed further metastasis to occur given the low efficacy of gemcitabine chemotherapy. Interestingly, a phase 1 clinical trial with FOLFIRINOX and Saridegib showed promising activity with a reported response rate of 67% but continuation of the trial was stopped and further development of the compound was halted [48].

Notch signaling is a highly conserved pathway involved with neurogenesis regulation during embryonic development and plays a role in the development and progression of pancreatic adenocarcinoma. The exact molecular mechanisms underlying how the notch pathway is involved in the pathogenesis have not been fully identified but there is cross talk between notch and other signaling pathways including, MEK/ERK, Hh, Wnt and others [49,50,51]. A novel strategy to target pancreatic cancer is to inhibit γ-secretase, which activates Notch. γ-secretase inhibitors (GSIs) have been shown in pancreatic cancer to inhibit cell growth, migration and invasion [52] by blocking epithelial–mesenchymal transition and suppressing pancreatic cancer stem cells (CSCs) [53]. Notch ligand delta like ligand 4 (DLL4) has been shown to be overexpressed in tumor cells leading to activation of notch signaling in CSC [54]. Demcizumab, a DLL4 inhibitor, potentially reversed chemotherapy resistance by targeting CSCs. The YOSEMITE study was a large randomized placebo controlled study comparing gemcitabine, nab-paclitaxel with or without demcizumab. Unfortunately, the study did not meet its primary endpoint [55].

Janus kinase/signal transducer and activator of transcription (JAK/STAT) has been shown to be involved in cancer development and progression. The JAK/STAT pathway facilitates signal transduction from multiple tyrosine kinase receptors and mediates inflammatory response in host and tumor tissue. STAT3 is required for pancreatic ductal adenocarcinoma progression in tumors harboring activated KRAS. Ruxolitinib—a potent JAK1/JAK2 inhibitor—is approved to treat hydroxyurea intolerant/refractory PV, high-risk myelofibrosis (MF) which includes: primary MF, post–polycythemia vera MF and post-essential thrombocythemia MF. Ruxolitinib, in combination with capecitabine, did not show a difference in PFS or overall survival during interim analysis and so the phase 3 trial was stopped [56]. STAT3 inhibition has been shown to decrease tumor growth in pancreatic cancer mouse models [57]. The stem cell pathway inhibitor that inhibits STAT3 transcription, napabucasin has shown efficacy in early trials [58]. This first-in-class cancer stemness inhibitor targets STAT3 driven gene transcription and spherogenesis of the cancer stem cell. In the phase Ib/II trial, 71 patients enrolled and there were no unexpected toxicities. The most common adverse events were diarrhea, nausea, vomiting and electrolyte abnormality. Early data showed a median progression free survival of >7.1 months and a median overall survival of >10.4 months. Based upon these encouraging results, it is now being evaluated in a phase III study in combination with gemcitabine and nab-paclitaxel (NCT02993731). Other approaches are on-going as well. AZD-1950 is an antisense STAT3 inhibitor is being evaluated in combination with durvalumab in a phase II trial that is currently actively recruiting patients (NCT02983578).

## 7. Targeting DNA Damage Repair Mechanisms

A majority pancreatic cancer cases are sporadic with 5–10 percent being familial in etiology. The most common genes associated with familial pancreatic cancer include BRCA1/2 and PALB2. BRCA2 mutations increases the risk of pancreatic cancer by 3.5-fold [59] and account for up to 17% of familial cases [60]. BRCA1 and BRCA2 are tumor suppressors genes involved in the repair of DNA. Its protein products form the complex necessary to repair DNA double strand breaks. In the setting of BRCA mutations, the poly-ADP ribose polymerases (PARP) of the base excision repair pathway is utilized for DNA repair making it an excellent therapeutic target. O’Reilly, et al. conducted a phase I/II clinical trial of gemcitabine, cisplatin and veliparib. In the dose escalation phase 1 study, 17 patients were accrued and 9 had a BRCA mutation. In this small cohort, an impressive 66% objective response rate was observed with a disease control rate of 88%. The dose limiting toxicities were mainly hematologic and larger studies are on-going with this promising treatment [61].

Acquired somatic mutations in homologous recombination genes are estimated to be between 3.9–35% [62,63]. These sporadic mutations can result in a BRCAness phenotype which is defined as “traits that usually occur in BRCA1/2 mutation carriers but are also present in some sporadic cancers” [64]. This concept has been proven recently in ovarian cancer patients with the phase III NOVA study. Niraparib, a PARP-1/2 inhibitor, showed benefit across patients and is approved for platinum sensitive relapsed ovarian cancer. The PFS benefit was most dramatically seen in germline BRCA mutated tumors and non-mutated BRCA that were identified to have homologous recombination deficiency (HRD) compared to placebo [65]. The HRD score is a composite of loss of heterozygosity, telomeric allelic imbalance and large-scale state transitions and measures genomic instability reflecting tumor homologous recombination DNA repair deficiency. This has been used as a predictor for response to platinum based chemotherapy and PARP inhibitors. SWOG S1513 is a second line trial randomizing patients with refractory metastatic pancreatic cancer to FOLFIRI +/− ABT888. Recently, this trial was closed by the data and safety monitoring committee but additional analysis is being done evaluating response based upon HRD score [66].

## 8. Immunotherapy

Early trials with checkpoint inhibitors in this disease have proven to be largely unsuccessful. Only in the small population of patients with MSI high tumors are there impressive responses seen [67,68]. Mechanisms of resistance may be due to the microenvironment of the stroma with regulatory T cells or myeloid derived suppressor cells to tumor derived expression of indoleamine-2,3-dioxygenase (IDO). This has led to numerous trials attempting to target the immune system to cancer.

### 8.1. Cancer Vaccines

GVAX is a cancer vaccine composed of tumor cells genetically modified to secrete granulocyte-macrophage colony-stimulating factor. In a previous trial combining GVAX with CRS 207, a recombinant Listeria base cancer vaccine expressing human mesothelium, results were promising. In an early phase study presented by Dr. Le [69], 90 patients were randomly assigned in the 2-1 ratio to 2 doses of cyclophosphamide and GVAX followed by 4 doses of CRS to 7 or 6 doses of cyclophosphamide and GVAX every 3 weeks. The primary endpoint was overall survival. At a pre-specified protocol analysis, patients receiving at least 3 doses of treatment had an improvement in overall survival of 9.7 versus 4.6 months. An enhanced mesothelium specific CD8 T-cell response was observed in patients with a longer survival. This prime boost approach appeared to be promising however in a larger trial conducted in the refractory population, the combination of GVAX and CRS 207 had a shorter survival compared to cytotoxic chemotherapy. Interestingly the CRS 207 alone arm had a better survival compared to the combination. Currently there are trials underway testing this vaccine approach earlier in treatment when the immune system is more robust.

Algenpantucel-L is a cancer vaccine comprised of irradiated allogeneic pancreatic cancer cells transfected to express murine alpha-1,3-galactosyltransferase. The phase 2 studies appear to be promising with twelve-month median overall survival rates of 86%. However, in the IMPRESS trial, which is a phase 3 randomized study of the vaccine in combination with chemotherapy with or without radiation therapy in patients with resected disease, there was no survival benefit seen [70].

### 8.2. Adoptive Cell Therapy

T cells placed central role in cell-mediated immunity and are part of the adaptive immune system. Cytotoxic T cells are designed to destroy virus infected cells were tumor cells and recognized her targets by binding to antigens associated with MHC class I molecules. The naive T cells will expand and differentiate into memory and effector T cells after being presented. However, this is dependent upon the T cells finding the antigen on the tumor. There are many mechanisms by which the tumor evades the immune system and adoptive cell therapy attempts to overcome this. T cells are collected from the patient and genetically modified to recognize the target. They are then expanded ex-vivo and then reinfused into the patient. This therapy is very individualized which is very labor and time intensive. This poses a challenge in patients that have very aggressive disease. There have been some promising results. Rosenberg’s group recently published on adoptive cell therapy using ex-vivo expanded tumor infiltrating lymphocytes. He identified polyclonal CD8 positive T cells against mutant K-RAS G12D and tumor infiltrating lymphocytes obtained from patient with metastatic colorectal cancer. After ex-vivo expansion and reinfusion to the patient, they observed progression of lung metastasis. Upon follow-up, one lesion had progressed and tumor analysis revealed loss of chromosome 6 haplotype that coded for the HLA C*08:02 class I MHC molecule [71]. This provided the tumor a mechanism for evasion of the immune system. This poses a challenge that despite successful initial treatment, mechanisms of resistance eventually developed. To repeat the process of adoptive cell therapy and identifying another neoepitope to target may not be feasible for an aggressive cancer but this is an encouraging step in targeting immunotherapy.

## 9. Conclusions

Pancreatic ductal adenocarcinoma continues to have a poor prognosis. Identifying ways to improve survival is a critical necessity. We are making progress as we learn more about the genomic alterations in pancreatic cancer. The Australian group performed an integrated genomic analysis of 456 pancreatic ductal adenocarcinomas and found 32 recurrently mutated genes aggregated into 10 pathways. By expression analysis they identified four subtypes of pancreatic cancer: squamous, pancreatic progenitor, immunogenic and aberrantly differentiated endocrine and exocrine. Each subtype has a unique molecular profile. As we learn more about each of these subtypes, we will likely see differences in growth, proliferation and metastasis. There are currently ongoing clinical trials trying to target different aspects of these pathways. For pancreatic cancer, we may not be looking for a driver mutation but rather a collection of pathways that may be activated for particular subtype. Hopefully this will help in the design of future trials with a multipronged approach that may or may not include a cytotoxic backbone. This would be a paradigm shift from our standard design of clinical trials adding drug X to the currently approved regimen. Gemcitabine and nab paclitaxel or FOLFIRINOX already has significant toxicities and it is challenging to add additional drugs to this backbone. Whether or not it is through utilizing cytotoxic chemotherapy, small molecule inhibitors, monoclonal antibodies or programming adaptive immunity, we are entering a new era of precision medicine which will improve survival for patients.

## Figures and Tables

**Table 1:** Select trials targeting RAS.

Drug	Mechanism of Action	Clinical Trial	Population (*n*)	Comparison	OS	PFS
Tipifarnib [26]	farnesyltransferase inhibitor	Phase III	Treatment naïve Advanced or metastatic pancreatic adenocarcinoma (*n* = 688)	Gemcitabine + tipifarnib or placebo	193 vs. 182 days	112 vs. 109 days
Salirasib [29]	prenylated protein methyltransferase inhibitor	Phase I	Treatment naïve metastatic pancreatic cancer gemcitabine plus salirasib (*n* = 19)	none	6.2 months	3.9 months

OS = overall survival; PFS = progression-free survival.

**Table 2:** Select trials targeting downstream of RAS.

Drug	Mechanism of Action	Clinical Trial	Population (*n*)	Comparison	OS	PFS	ORR
Selumetinib [33]	MEK 1/2 inhibitor	NCT00372944 Phase II	Metastatic pancreatic adenocarcinoma who had failed first line gemcitabine (*n* = 70)	Capecitabine	5.4 vs. 5.0 months (HR 1.03; 80% CI 0.68–1.57; *p* = 0.92)	2.1 vs. 2.2 months (HR 1.24; 80% CI 0.88–1.75; *p* = 0.41)	
Trametinib [34]	MEK 1/2 inhibitor	Phase II	Metastatic adenocarcinoma of the pancreas with no prior therapy for metastatic pancreatic adenocarcinoma in combination with gemcitabine (*n* = 160)	Placebo	8.4 vs. 6.7 months (HR 0.98; 95% CI 0.67–1.44; *p* = 0.453)	16.1 vs. 15.1 weeks (HR 0.93; 95% CI 0.65–1.34; *p* = 0.349)	22 vs. 18%
Selumetinib + Erlotinib [35]	MEK 1/2 inhibitor + EGFR inhibitor	NCT01222689 Phase II	Locally advanced or metastatic pancreatic adenocarcinoma with one line prior therapy (*n* = 46)	None	7.3 months (95% CI, 5.2–8.0)	1.9 months (95% CI, 1.4–3.3)	0%
Selumetinib + MK-2206 [32]	MEK 1/2 inhibitor + AKT inhibitor	SWOG S1115 Phase II	Metastatic pancreatic adenocarcinoma (*n* = 137)	mFOLFOX	3.9 vs. 6.7 months (HR 1.37; 95% CI 0.90–2.08; *p* = 0.15)	1.9 vs. 2.0 months (HR 1.61; 95% CI 1.07–2.43; *p* = 0.02)	2 vs. 7% (*p* = 0.21)

ORR = objective response rate.
